# Visual and Imagery Magnitude Comparisons Are Affected Following Left Parietal Lesion

**DOI:** 10.3389/fpsyg.2017.01622

**Published:** 2017-09-19

**Authors:** Yarden Gliksman, Sharon Naparstek, Gal Ifergane, Avishai Henik

**Affiliations:** ^1^Department of Psychology and Zlotowski Center for Neuroscience, Ben-Gurion University of the Negev Beer-Sheva, Israel; ^2^Department of Rehabilitation, Soroka University Medical Center Beer-Sheva, Israel; ^3^Department of Neurology, Soroka University Medical Center Beer-Sheva, Israel

**Keywords:** acalculia, IPS, working memory, size congruity, mental manipulation, distance effect, magnitude comparison

## Abstract

We describe Jane Dow (JD), a young right-handed female with acalculia following a cerebral infarction in the left intraparietal sulcus. We investigated automatic processing of different types of magnitudes that were presented visually or through imagery. We employed the size congruity task and the mental clock task that differ in stimuli presentation and in working memory load. In the size congruity task, for physical comparisons, JD presented a lack of facilitation effect, suggesting a deficit in the automatic processing of numerical values. In the mental clock task, JD performed as accurate as controls did but much slower. In both tasks, JD presented a steeper distance effect compared to controls, suggesting a deficit in a domain-general comparison process. Our findings present an atypical pattern of magnitude processing following a left parietal lesion that appears not only for visually presented stimuli but also for imagery-based magnitudes. These finding support recent theories suggesting different types of magnitudes are interconnected with each other.

## Introduction

The current study focused on numerical abilities and comparative judgment in a young patient following an infarct in her left parietal cortex. Basic numerical operations such as comparison of visual and imaginary magnitudes were examined in order to explore the role of the left parietal cortex in magnitude processing.

The parietal cortex and more specifically, the intraparietal sulcus (IPS), plays an important role in magnitude processing. Previous studies found that the IPS is involved in various aspects of number processing (Dehaene and Cohen, 1995, 1997) and more specifically, in magnitude comparison and enumeration (Piazza et al., 2002; Ansari and Dhital, 2006; Ansari, 2007). Damage to the left intraparietal lobe was suggested to lead to primary acalculia (Takayama et al., 1994), a condition in which a person presents deficits in numerical abilities that cannot be attributed to other sequels of cerebral disturbances, such as aphasia, memory or attentional disorders and dementia (Kaufmann et al., 2013). Neuropsychological studies with patients following brain lesions support this assumption. For example, Delazer and Benke (1997) reported on JG, a 56-year-old patient, who underwent surgery in her left IPS due to a tumor. Following the surgery, her calculation abilities (mostly multiplication) and the use of arithmetic procedures were intact, but she presented a severe deficit in conceptual knowledge of math, such as understanding the meaning of arithmetic operations (e.g., the patient could not explain the meaning of multiplication). JG could carry out number comparisons (decide if a number was smaller or larger than 5), and could count from 1 to 20, but she was slower compared to controls in both tasks. Delazer et al. (2006) reported on HR, a 62-year-old patient who suffered from a rare syndrome of dementia, posterior cortical atrophy (PCA), with cerebral deterioration to bilateral posterior parietal regions. The patient presented specific numerical deficits in tasks that required access to the internal representation of numbers, such as semantic facts, estimation, number comparison and number bisection (deciding if a number was between two given numbers or not). Furthermore, she presented a larger distance effect compared to controls (Delazer et al., 2006). The distance effect occurs during number comparison and is indicated by a decrease in response time as the distance between the digits increases (Moyer and Landauer, 1967). This effect serves as a marker for the mental representation of numbers (Dehaene and Cohen, 1995), and a larger distance effect might suggest a less distinct representation of magnitudes (Holloway and Ansari, 2008). These two case studies suggest that the parietal lobe indeed plays an important role in numerical processing, and lesions in this area might lead to deficits in a wide range of mathematical skills and abilities (van Harskamp and Cipolotti, 2001).

The parietal lobes, and more specifically the IPS, also plays an important role in working memory (Marshuetz et al., 2006; van Dijck and Fias, 2011; Attout et al., 2014), related to a limited capacity system which temporarily maintains and stores information (Baddeley, 2003). The three-component model is a popular model that divides the system into a modality-dependent control component (i.e., central executive), assisted by two modality-specific slave components: the phonological loop and the visuo-spatial sketchpad. The phonological loop processes auditory cues and language, and maintains serial orders whereas the visuo-spatial sketchpad processes visual and spatial information. The central executive is in charge of controlling processing through coordination of multiple cognitive functions and its efficiency can be studied through capacity measures such as span (i.e., the number of items a subject can repeat) (Baddeley, 2003; McCabe et al., 2010). Interestingly, working memory capacity is restrained by the speed of processing of information. In a study on cognitive decline during aging, Salthouse (1996) suggested that slower processing speed limits the time allowed for rehearsals of information and search during retrieval; limited time also affects the amount of information that can be presented simultaneously, leading to a smaller capacity overall. Imaging studies and converging evidence from lesion studies suggest the phonological loop involves the left temporo-parietal cortex, whereas visual working memory relies mostly on the right hemisphere. Since both working memory and magnitude processing seem to rely on shared cortical regions, several studies examined the links between these functions. These studies suggest working memory plays a crucial role in magnitude processing, specifically through maintenance of serial order through the phonological loop (Doricchi et al., 2009; van Dijck and Fias, 2011; Attout et al., 2014).

One of the widely applied tasks to study numerical comparisons is the size congruity task (Henik and Tzelgov, 1982). In this comparison task, participants are presented with two digits that differ in numerical or physical size and are asked to decide which digit is larger (physically or numerically, in different blocks). Since in each comparison participants are asked to attend to one dimension only (physical or numerical), processing of the irrelevant dimension will suggest it is activated automatically (Tzelgov et al., 1992). Numerical and physical sizes can be congruent (the numerically larger digit is also physically larger, e.g., 3 5), incongruent (the numerically larger digit is physically smaller, e.g., 3 5) or neutral (the digits differ only in one dimension—for the physical task, they are similar in numerical size, e.g., 3 3; for the numerical task they are similar in physical size, e.g., 3 5). Due to the correspondence between the physical and numerical aspects of the stimuli, responses to congruent trials are faster than to neutral trials and these in turn are faster than to incongruent trials. The benefit of the congruent trials over the neutral trials is referred to as the facilitation component; and the cost of the incongruent trials in relation to the neutral ones is referred to as the interference component (MacLeod, 1991). Importantly, previous studies suggest these two components occur at different stages of processing and reflect different cognitive functions. In an ERP study, facilitation was related to both earlier perceptual and later response selection stages, whereas interference was related only to the later response selection stage (Szűcs and Soltész, 2007). This differentiation was also described in a study examining the developmental trajectory of size congruity. Specifically, in the physical task, interference appeared in children at the end of first grade, whereas facilitation appeared in children only in the third grade. The lack of facilitation at younger ages was attributed to a less automatic representation of numerical magnitudes (Rubinsten et al., 2002).

The effect of parietal brain lesions on automaticity of numerical values and on numerical distance was addressed in a study by Ashkenazi et al. (2008). The authors reported on AD, a 67-year-old patient with a left IPS injury who presented a deficit in perception and manipulation of quantity. In order to test his basic numerical abilities, the size congruity task was employed. In the numerical comparison, AD showed a congruity effect similar to controls, suggesting intact automaticity of physical sizes. As for the distance effect, he presented a steeper effect. In the physical comparison, AD showed a different pattern, resulting from a lack of facilitation (specifically, response times were slower to congruent trials compared to neutral trials). These findings led the authors to conclude that the IPS is necessary for the processing required in size congruity comparisons.

Aside from number comparisons, researchers have also examined comparisons of other magnitudes such as objects (Moyer, 1973; Paivio, 1975), object names (Rubinsten and Henik, 2002; Setti et al., 2009), lengths and brightness (Cohen Kadosh and Henik, 2006) and mentally constructed magnitudes (Paivio, 1978). For example, in the mental clock task (Paivio, 1978), participants are presented with pairs of times (e.g., 2:30 and 5:30) and are asked to indicate, using key presses, which time involves a larger angle. Participants are instructed to carry out the task while imagining clock faces and mentally comparing the imagined angular sizes created by the clock hands. Aside from mental imagery abilities, the mental clock task enables exploring the distance effect for imagined stimuli. Paivio presented time pairs with angular size differences of 30, 60, 90, 120, or 150° between the clock hands, and hypothesized that, similar to other magnitude comparisons (objects, numbers, etc.), reaction time (RT) would decrease as the angular difference between clocks would increase. For example, in the time pair 3:20 and 2:25, the hour and minute hands form angles of 30 and 90°, respectively; thus, the larger angle appears at the 2:25 time, and the angular difference between the two clocks is 60°. Indeed, there was a distance effect for the imagined clocks, suggesting a similar process for comparison of visual and imagined stimuli. This similarity between imagined and visually presented information was also found in a functional magnetic resonance imaging (fMRI) study where an imagery task (i.e., mental clock) and a perceptual task (i.e., visual comparison) revealed similar brain activation, specifically in the posterior parietal cortex (PPC), suggesting a common neural substrate underlies the spatial comparison of imagery and perceptual stimuli (Trojano et al., 2000). Analysis of the temporal sequence of brain activation using fMRI (Formisano et al., 2002) revealed a different role for the left and right hemispheres while carrying out the mental clock task. Specifically, the left PPC was activated during earlier stages whereas the right PPC was activated both during earlier and late stages, but was more prominent during late stages of processing. The authors suggested this asymmetry might explain the specific role of each hemisphere in image construction and analysis. Namely, early construction of a mental image is subserved mainly (but not exclusively) by the left PPC, whereas later image analysis (comparison) is subserved mainly by the right PPC. Analysis of participants’ RTs was given as further support for this assumption. It was shown that responses were faster when participants used their left hand and imagined clock hands were in the right hemifield, suggesting earlier left activation (image construction) and later right activation (angle comparison and response execution) will result is faster RTs for this presentation compared to the opposite (i.e., right-hand response and left hemifield presentation). This hemispheric specialization was also supported by a combined fMRI repetitive transcranial magnetic stimulation (rTMS) study (Sack et al., 2002). Here, performance in the imagery task was affected only by rTMS to right, and not left, parietal regions suggesting right parietal regions can compensate for left suppression but not vice versa. Thus, the early process of image generation, hypothesized to rely mainly on left parietal regions, might be carried out by a larger bilateral network whereas the later comparison and analysis stages indeed rely mainly on right parietal regions.

### Current Study

We employed both a magnitude comparison task and a mental imagery task to assess comparison abilities in a patient who suffered from primary acalculia following left parietal brain damage. We assessed visual magnitude comparison using the size congruity task, and imagery magnitude comparison using the mental clock task. These two tasks differ in the stimuli presented and thus, in the working memory load. In the size congruity task, participants are presented with visual symbols and are asked to compare between their magnitudes. Since symbols are present during the task, no image construction or maintenance is needed, suggesting little involvement of working memory in the task. In the mental clock task, however, participants are presented with auditory descriptions for which they need to construct magnitudes. These mentally constructed magnitudes must then be maintained in order for the comparison process. Thus, the mental clock task posits a higher load on the working memory system. Importantly, in both tasks participants are asked to *compare magnitudes*, enabling us to examine the similarities and differences between comparison of different magnitude notations (e.g., visual and auditory; presented and constructed). Moreover, in both tasks the numerical distance between the values to-be-compared is manipulated, enabling us to examine whether the distance effect is modulated by the format. Finally, by testing these tasks on a patient following a focal brain lesion, we can examine the extent of the parietal lesion over two different magnitude comparisons.

Following the work of Ashkenazi et al. (2008), we expected to replicate previous findings and hypothesized the patient would present deficits in numerical processing as revealed in the size congruity task. Namely, in the physical comparison we expected a congruity effect composed mostly of an interference component and a lack of a facilitation component. As for the distance effect, similar to previous findings on comparison tasks, in the size congruity task, we expected a larger distance effect compared to controls (Delazer and Benke, 1997; Ashkenazi et al., 2008). In the mental imagery task, considering the specific role of the left and right hemispheres in carrying out the task, we hypothesized the intact right PPC would compensate for the lesioned left PPC, enabling the patient to carry out the task as accurately as controls (Formisano et al., 2002; Sack et al., 2002). However, since early image construction, as well as working memory abilities, might have been affected by the left parietal lesions, we expected the patient to carry out the task slower than controls. Finally, if indeed visual (perceptual) and imagined stimuli tap the same neural networks (Trojano et al., 2000), we expected the patient would present a steeper distance effect in the mental clock task as well. Whereas previous studies compared the distance effect between numerical and non-numerical stimuli (Cohen Kadosh et al., 2005; Holloway and Ansari, 2008), to the best of our knowledge, no other study has examined the distance effect in *visual* and *imagined* magnitudes in the same study. A larger distance effect in both tasks would suggest a deficit in the representation of both perceptual and imagined magnitudes. This in turn, would strengthen the notion of a core representation of magnitude, which is activated regardless of the type of stimuli presented (Walsh, 2003; Szűcs and Soltész, 2007; Henik et al., 2012).

Finally, most patient studies at the individual (Delazer et al., 2006; Ashkenazi et al., 2008) or group level (Baldo et al., 2010) have tested elderly patients. Thus, cognitive decline due to aging might have contributed or facilitated some of the deficiencies reported. Here we report on a relatively young patient, enabling us to explore the effect of a focal lesion while eliminating any cognitive deterioration due to aging processes. Namely, any abnormal findings could be accounted for solely by the lesion.

## Materials and Methods

### Case Description

**FIGURE 1:**
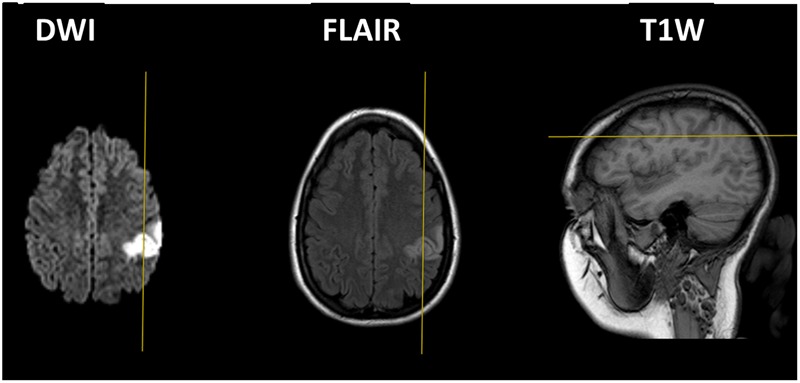
Magnetic resonance imaging (MRI) scans in DWI **(left)**, flair **(middle)** and T1W **(right)**, demonstrating an infarct in the left IPS. The right side of the picture refers to the left side of the brain. The left and middle pictures present horizontal sections and the right picture presents a sagittal section.

JD (henceforth, JD; the patient’s name has been changed to protect her anonymity) is a patient in her early 20’s, otherwise healthy, right-handed female [as assessed by the Edinburgh Handedness Inventory (Oldfield, 1971)]. She was admitted to the Department of Neurology at Soroka University Medical Center due to mild right-sided weakness and transient speech disturbances. The neurological examination on admission revealed pronation drift of the right hand and right-sided hypoesthesia. A bedside cognitive screening using the Montreal Cognitive Assessment (MoCA) (Nasreddine et al., 2005) revealed intact orientation, visuo-spatial, and language abilities, and a deficit in delayed memory and working memory, specifically in tasks involving digits and mathematical operations, leading to an overall lower-than-average performance (MoCA = 25/30, *z* = -1). Aside from these deficits, there were no signs for finger agnosia, left-right disorientation or agraphia, and thus no signs of Gerstmann’s syndrome (Rusconi et al., 2009). An MRI scan carried out upon admission (**Figure 1**) demonstrated an infarct in the left parietal cortex in the area of the IPS. Importantly, the lesion was purely cortical with no involvement of major white matter tracts. The abnormal high signal on diffusion-weighted imaging (DWI) indicated restriction of water molecule movement secondary to cytotoxic edema, which in the given clinical history, was typical of an acute infarct. The lesion was clearly seen in both Flair and T1 sequences. CT (computed tomography) angiography did not disclose any vascular abnormality. Cardiac evaluation using both transthoracic and transesophageal echocardiography was unremarkable. No hematological abnormality was detected. She was diagnosed as suffering from cryptogenic stroke, treated by aspirin, and referred to rehabilitation.

JD completed 12 years of formal education and was working as a secretary. Her arithmetic abilities prior to the incident were average, as can be inferred from her grades in math both in high school and in the Psychometrics Entrance Test (PET, a national test used to screen applicants in institutions of higher education). JD gave written informed consent to participate in the study, which was previously approved by the local Helsinki committee.

### Control Participants

Since JD was relatively young, her performance was compared to control groups of students studying at Ben-Gurion University of the Negev. The control groups were different for the different tasks. The arithmetic battery, sustained attention, and reading tasks were carried out on 52 control participants who were part of a larger group of control participants in a norm study (24 females; age: 23.2 ± 1.7 years old). The size-congruity task was carried out on 16 control participants (11 females; age: 24 ± 1.7 years old). The mental clock task was carried out on 25 control participants (11 females; age: 24.9 ± 1.8 years old). Participants were paid in return for their participation (about $8 per hour) or received a course credit. This study was carried out following the guidelines of the protocol approved by the university’s ethics committee.

### Procedure

The study was carried out in two sessions. The first session took place 1 week after JD’s admission, that is, 1 week after her infarct. During this session, neuropsychological (i.e., intelligence, working memory, attention and reading abilities) and arithmetic abilities were tested. The second session took place 3 months after the infarct. During this session, basic numerical and magnitude comparison abilities (i.e., size congruity task and mental clock task) were assessed.

### Neuropsychological Tests

Intelligence was measured by Raven’s Progressive Matrices (Raven and Court, 1998). Sustained attention was assessed using the CPT (continuous performance task), following the task reported in Shalev et al. (2011). Working memory was examined by applying both the WAIS-III^HEB^ (Wechsler Adult Intelligence Scale – Third Edition, Hebrew Version) and the WMS-III (Wechsler Memory Scale) relevant subtests (WAIS-III^HEB^: arithmetic, digit span and letter-number sequencing; WMS III: spatial span, letter-number sequencing). Reading abilities were measured by a text-reading test, and phonological awareness was measured using a non-word reading test (taken from the Hebrew reading and writing diagnostic battery “Alef and Taf”, Shany et al., 2006).

### Arithmetic Battery

We used a paper and pencil arithmetic battery based on the one reported by Ashkenazi and Henik (2010a), with a few changes. The battery included 13 subtests in two parts: (a) number comprehension and production; and (b) calculation. The changes from the original battery included adding additional items to each subtest, and exclusion of two subtests (recognition of numeral place value in written numbers; non-numerical series progression). For more information about the subtests, see Supplementary Materials.

### Cognitive Tasks

We used an IBM computer in all tasks. The tasks were programmed in E-Prime 1.0 (Schneider et al., 2002).

#### Size Congruity Task

We used a task a similar to the one applied by Ashkenazi et al. (2008). Each trial was composed of the presentation of two single digits—one on the right side of the computer screen and the other on the left side—at an equal distance from the center. The two digits differed in their physical sizes and numerical values. In different blocks, participants were instructed to indicate by a key-press, which digit was numerically or physically larger. Stimuli were composed of congruent (e.g., 3 5), incongruent (e.g., 3 5), and neutral (e.g., 3 5 for numerical comparison, and 3 3 for physical comparison) conditions. There were three numerical distances: 1 (with pairs 1 2, 3 4, 6 7, 8 9), 2 (with pairs 1 3, 2 4, 6 8, 7 9), and 5 (with pairs 1 6, 2 7, 3 8, 4 9). The physical size of the larger digit was 0.7 cm × 1 cm and the physical size of the smaller digit was 0.5 cm × 0.7 cm. The numbers were presented at the center of the screen in an 11 cm × 8.5 cm field. Each block was composed of 288 trials: 3 congruency × 3 numerical distances × 4 different pairs of digits for each distance × 2 sides (larger digit left∖right) × 4 repetitions of each trial. Each trial began with a fixation asterisk for 300 ms. Once the fixation disappeared, two digits appeared at the center of the screen until the participant’s response. The next trial began 1,000 ms after the response. RT was measured in milliseconds from target onset until the participant’s key-press.

#### Mental Clock

The mental clock task was based on the task presented by Paivio (1978) and modified by others (Formisano et al., 2002; Sack et al., 2002). Participants were asked to imagine two analog clock faces based on acoustically presented times, and to choose the one in which the clock hands formed a larger angle. Participants were instructed to press a left key if the first time created a larger angle and a right key if the second time created a larger angle. Prior to the imagery task, participants carried out 9 trials with visually presented analog clocks, followed by 12 acoustic practice trials in which feedback was given to assure familiarization with the stimuli and response. There were four angular differences: 30, 60, 90, and 120°, repeated 12 times each, resulting in 48 experimental trials. Trials were balanced for the side of the clock hands to be imagined (i.e., left, e.g., 19:00 21:00; right, e.g., 13:00 13:30; and mixed e.g., 15:30 20:30) and for the response hand (right, left). Each trial began with a fixation dot for 1,000 ms, after which the two acoustic stimuli were presented for 2,400–4,000 ms, and then the participant’s response was given. This was followed by a 4,000 ms interval before the next trial began. RT was measured in milliseconds from the acoustic target’s offset until the participant’s key-press.

## Results

In order to compare JD’s performance to that of controls, we employed the Crawford analysis (Crawford and Garthwaite, 2004; Crawford et al., 2004). This analysis was designed in order to test whether an individual did or did not come from a population of controls. Importantly, this analysis is highly appropriate in small samples (less than 60) and is commonly used when comparing the performance of single case patients to controls (Ashkenazi et al., 2008; Cavina-Pratesi et al., 2010).

### Neuropsychological Test

In an intelligence test, JD performed similar to controls. Overall, working memory abilities were between the average to lower-than-normal ranges. However, a closer examination of the subtests suggests some working memory abilities were compromised. For examples, although JD’s performance in the arithmetic test was within the average range, using a paper and pencil (thus, compensating for possible working memory deficits) enhanced her abilities (an addition of 3 correct responses leading to a scaled score of 15). It also seems that performance was mostly affected in the span tests (both digits and spatial) with lower performance in working memory maintenance (i.e., digits/spatial sequence forward) compared to working memory manipulation (i.e., digits/spatial sequence backward) (Snyder et al., 2015). The performance in an attention test revealed significantly slower responses but higher accuracy rates compared to controls. Accuracy rates in reading abilities and phonological awareness were similar to those of controls (see **Table 1A**).

**Table 1A:** Neuropsychological tests.

	Control	JD	*t*	*p*
Working memory index scaled score (WAIS-III^HEB^, WMS)		92, average 83, low-normal^a^		
Arithmetic		12^b^		
Letter-number sequencing		8^b^		
Digit span		6^b^ (forward = 4, *Z* = -2.2, backward = 4, *Z* = -0.7)		
Spatial span		6^b^ (forward = 5, backward = 4)		
Raven	Percentile 42 (5)	Percentile 40	-0.396	0.347
CPT (RT)	0.419 (0.38)	517	2.573	0.013^∗^
CPT (ACC)	93 (0.09)	96	33.017	0.0001^∗^
Reading test (RT)	103 (10.3)	99.5	1.154	0.127
Reading test (ACC)	99.5 (0.76)	99	0.652	0.259
Non-word (RT)	35 (3.1)	96	19.49	0.0001^∗^
Non-word (ACC)	94 (12.9)	80	-1.075	0.144

### Arithmetic Battery

#### Number Comprehension and Production

Jane Dow’s performance on all number comprehension and production tests was significantly slower than controls. Her accuracy rates were significantly lower in all subtests but one (comparing digits). Importantly, in the procedural knowledge subtest, JD could not perform the transpose equation subtest (i.e., copy an equation from a horizontal into a vertical presentation) (see **Table 1B**).

**Table 1B:** Arithmetic battery part A: number comprehension and production.

	Control	JD	*t*	*p*
Comparing digits (RT)	12 (2.3)	25	5.599	0.0001^∗^
Comparing digits (ACC)	100 (0.1)	100	0	0.5
Counting forward (RT)	52 (10.1)	86	3.334	0.001^∗^
Counting forward (ACC)	92 (0.7)	71	-29.716	0.0001^∗^
Counting backward (RT)	47 (10.9)	113	5.998	0.0001^∗^
Counting backward (ACC)	95 (0.6)	83	-19.81	0.0001^∗^
Serial order (RT)	81 (26)	253	6.553	0.0001^∗^
Serial order (ACC)	98 (0.6)	100	3.302	0.002^∗^
Comparing fractions (RT)	12 (3.5)	50	10.754	0.0001^∗^
Comparing fractions (ACC)	98 (0.5)	50	-95.09	0.0001^∗^
Verbal problems (RT)	204 (60)	360	2.575	0.006^∗^
Verbal problems (ACC)	88 (0.9)	75	-14.308	0.0001^∗^
Procedural knowledge (RT)	55 (16)	n/a		
Procedural knowledge (ACC)	87 (0.4)	0		

#### Calculation

**FIGURE 2:**
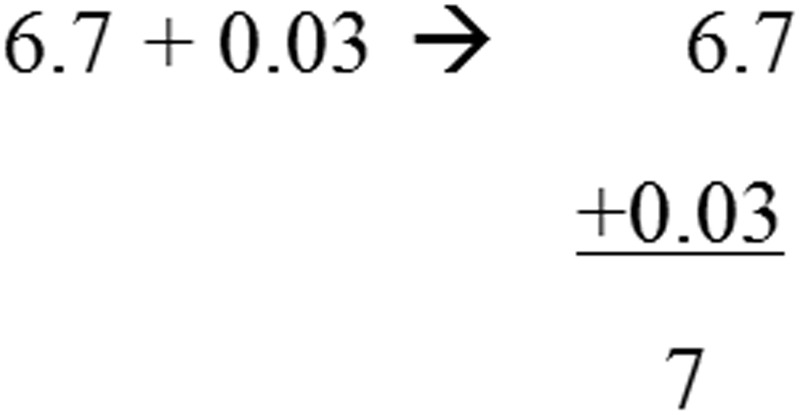
Example of an item requiring conceptual knowledge that was solved by JD. The original item appears on the left; JD’s solution appears on the right.

Again, JD performed significantly slower compared to controls in all subtests. In the simple and mixed operations, JD’s accuracy rates remained intact whereas in the more complicated subtests (i.e., comparing equations and vertical operations), JD presented low accuracy rates. For example, in solving a long division item, she reported the answer to 388:4 was 22; for the item 12,504:12, she reported the answer was 100; for the item 6.7+0.03, she reported the answer was 7 (see **Figure 2**). These erroneous responses might be accounted for either by a deficiency in the ability to keep the rule of place value of the numbers or by a wrong appreciation of the number magnitude (see **Table 1C**).

**Table 1C:** Arithmetic battery part B: calculation.

	Control	JD	*t*	*p*
**Simple operations**				
Addition (RT)	14 (4.3)	58	10.136	0.0001^∗^
Addition (ACC)	100 (0.3)	100	0	0.5
Subtraction (RT)	11 (3.2)	63	16.096	0.0001^∗^
Subtraction (ACC)	100 (0.2)	100	0	0.5
Multiplication (RT)	15 (5.6)	71	9.905	0.0001^∗^
Multiplication (ACC)	100 (0.4)	100	0	0.5
Division (RT)	16 (7.1)	98	11.44	0.0001^∗^
Division (ACC)	100 (0.5)	100	0	0.5
Mixed operations (RT)	56 (22)	241	8.329	0.0001^∗^
Mixed operations (ACC)	94 (1.6)	95	0.619	0.269
Decimals (RT)	79 (33.8)	n/a	–	–
Decimals (ACC)	90 (0.1)	0	–	–
Estimation (ACC)	80 (0.12)	77	-24.763	0.0001^∗^
Comparing equations (RT)	42 (13.7)	66	1.735	0.044
Comparing equations (ACC)	94 (0.9)	88	-6.603	0.0001^∗^
Vertical operation (RT)	147 (69)	313	2.383	0.01^∗^
Vertical operation (ACC)	81 (2.8)	75	-2.123	0.019^∗^

*Mean reaction time is in seconds; accuracy rate is in percentage; standard deviation is in parentheses; ^a^index scores are a standard score with a mean of 100 and standard deviation of 15; ^b^subtest scores are a standard score with a mean of 10 and a standard deviation of 3; *T-*test is one-tail probability; ^∗^significant difference between JD and controls according to Crawford et al. (2004). For more information about the subtests, see Supplementary Materials.*

### Cognitive Tasks

#### Size Congruity Task

Jane Dow’s accuracy rates were similar to those of controls, [JD: 98%; controls: 94%; two-tail probability, *t*(15) = 0.78, *p* = 0.45]. Similar to controls, her performance in numerical comparisons were slower than in physical comparisons. In both physical and numerical comparisons, congruent trials were responded to faster than incongruent trials were, leading to congruency effects. The Crawford analysis revealed that the congruency effects of JD and the controls were similar in both physical and numerical tasks [one-tail probability, *t*(15) = 0.23, *p* = 0.41 and *t*(15) = -0.17, *p* = 0.44, for the physical and numerical tasks, respectively]. Because we were mostly interested in the automatic processing of numerical information, we further analyzed the congruity effect in the physical task. Here, we found two marked differences in performance between JD and controls. First, whereas the controls presented a facilitation component [responses to congruent trials were faster than responses to neutral trials, *F*(1,15) = 4.92, *p* = 0.04, $\eta_{p}^{2}$ = 0.35], JD presented a lack of facilitation component. Moreover, replicating previous findings on patient AD, responses to neutral trials were faster than responses to congruent trials (Ashkenazi et al., 2008). Second, whereas both controls and JD presented an interference effect [responses to incongruent trials were slower than responses to neutral trials, *F*(1,15) = 8.27, *p* = 0.01, $\eta_{p}^{2}$ = 0.25], JD replicated previous findings on patient AD, and presented a significantly larger interference effect compared to controls [one-tail probability, *t*(15) = 1.75, *p* = 0.05] (see **Table 2**).

**Table 2:** Reaction time for physical and numerical comparisons in the size congruity task.

Group and task	Incongruent	Neutral	Congruent
**Physical**			
JD	637 (341)	581 (270)	608 (337)
Controls	416 (67)	403 (58)	393 (51)
**Numerical**			
JD	995 (395)	1077 (760)	933 (473)
Controls	564 (137)	522 (120)	495 (107)

*Reaction time in milliseconds; standard deviations in parentheses.*

**FIGURE 3:**
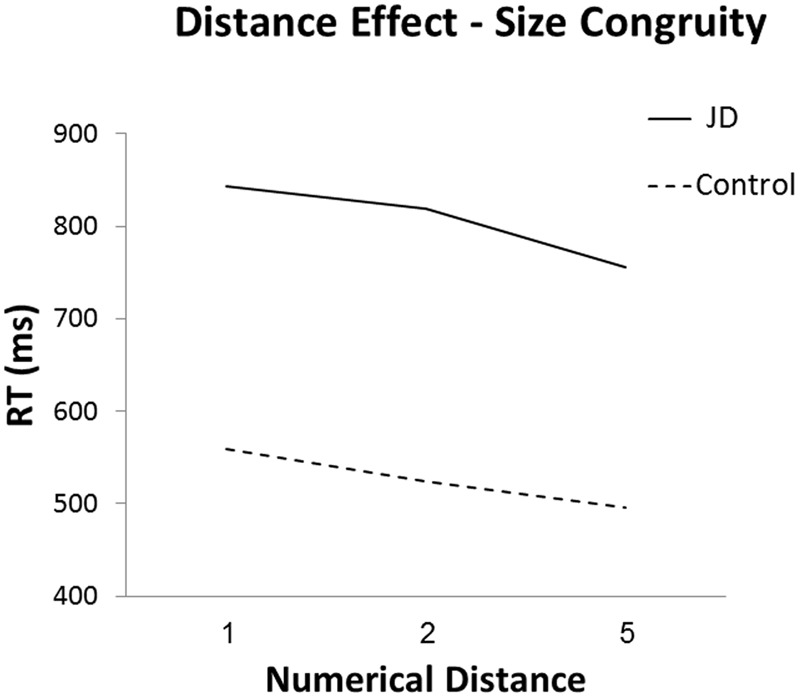
Reaction time in the numerical task as a function of numerical distance.

As for the distance effect, we analyzed the difference between mean RTs to adjacent distances (i.e., distance 1 and 2, distance 2 and 5) in the numerical task, and compared these differences between JD and the controls. We used the neutral trials in order to have a cleaner estimation of the distance effect. The Crawford analysis revealed that in both distances (distance 1 to 2 and distance 2 to 5), JD had a steeper slope compared to that of controls [distance 1 to 2: one-tailed probability, *t*(15) = 3.33, *p* = 0.018; distance 2 to 5: one-tailed probability, *t*(15) = 6.06, *p* < 0.001]; see **Figure 3**.

#### Mental Clock Task

**FIGURE 4:**
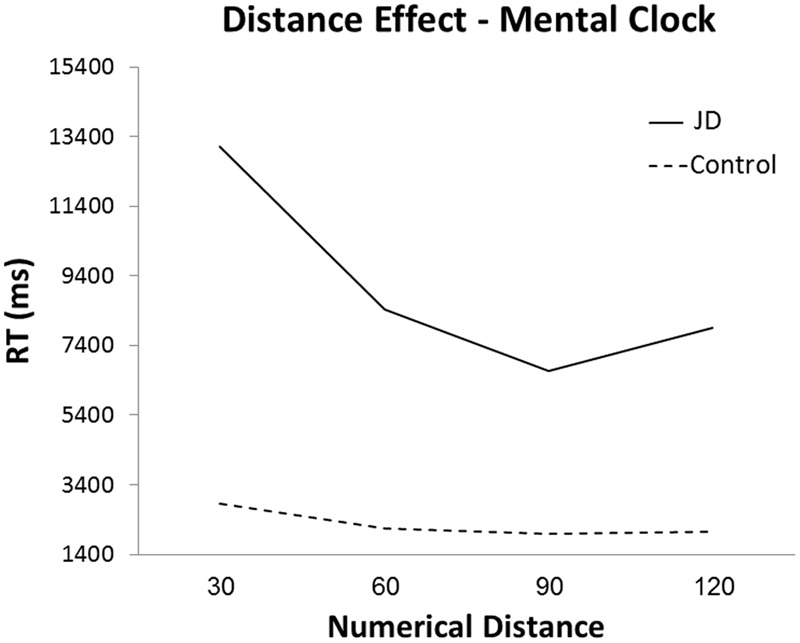
Reaction time in the mental clock task as a function of numerical distance in degrees.

In the mental clock task, JD performed as accurately as controls (88%), however, her performance was slower (mean RTs were: JD: 13,120, 8,422, 6,665 and 7,914 ms; controls: 2,859, 2,143, 1989, and 2,045 ms; for 30, 60, 90, and 120°, respectively, see **Figure 4**). Both JD and the controls presented a distance effect (RTs decreased as the distance increased) for the first two distances (30–60° and 60–90°), and a slight increase for the largest distance (90–120°). As can be seen in **Figure 4**, JD’s distance effect was much steeper compared to controls. A Crawford analysis revealed JD’s performance was different from controls in all adjacent distance pairs (distance 30–60°, *t* = 4.67, *p* < 0.001; distance 60–90°, *t* = 2.35, *p* = 0.014; distance 90–120°, *t* = -2, *p* = 0.035, one-tailed probability for all mentioned analyses). Similar to controls, JD’s responses were faster for clock hands imagined in the right visual hemifield. However, unlike controls, right-hand rather than left-hand responses were faster.

## Discussion

We reported the case of JD, a young patient who suffered from primary acalculia after a cerebral infarction in the left IPS. Our main findings are: (1) whereas intelligence and reading abilities remained intact, JD presented some impairments in working memory abilities, specifically in maintenance. (2) In an arithmetic battery, JD presented difficulties in accuracy and RTs. (3) In the size congruity task, in the physical comparison, where automatic processing of numerical size was examined, JD was slower, showed a lack of facilitation effect, and presented an increased interference effect. (4) In the mental clock task, JD’s accuracy rates were similar to those of controls, and performance was much slower compared to controls. (5) In both the size congruity task and the mental clock task, JD showed a larger distance effect compared to controls.

Jane Dow’s intact performance in intelligence and reading tests, along with some impairments in working memory abilities, and with the specific deficits in the arithmetic battery, is in accord with the existence of a focal cortical lesion in the left parietal lobe and more specifically, in the IPS. This neuropsychological profile supports the diagnosis of primary acalculia (Cipolotti et al., 1991) and fits with previous patient studies (Dehaene and Cohen, 1995; Delazer and Benke, 1997; Ashkenazi et al., 2008).

The lack of a facilitation effect and the steeper distance effect in the size congruity task replicate previous reports of patients with acquired brain damage in parietal regions. Specifically, patient AD showed both lack of facilitation and a larger distance effect (Ashkenazi et al., 2008), and patient HR showed a larger distance effect (Delazer et al., 2006). A lack of facilitation in physical comparison tasks appears when studying populations with immature numerical abilities. Rubinsten et al. (2002) examined the size congruity effect in 6- to 12-year-old children and described the developmental trajectory of the congruity effect and its components. They found that the interference component appears first, at the end of first grade, whereas the facilitation component appears only in third grade. A lack of facilitation was also described in students with developmental dyscalculia (Rubinsten and Henik, 2005). Dissociation between interference and facilitation in the size congruity task was also described in an ERP study, and was attributed to different stages of cognitive processing (Szűcs and Soltész, 2007). It was suggested that interference reflects an attentional process while facilitation reflects automaticity (Rubinsten et al., 2002; Ashkenazi and Henik, 2010b). Accordingly, the lack of a facilitation effect in the size congruity task might suggest a weaker automatic access to numerical representations. Alternatively, it has been suggested that when carrying out the size congruity task, participants generate codes for the magnitudes presented (more/less, large/small). In healthy individuals, these codes are aggregated and lead to facilitation of responses, while in brain-injured populations, the ability to aggregate the information is hampered, leading to dissociation between facilitation and interference (Ashkenazi et al., 2008; Cohen Kadosh et al., 2008a). Accordingly, the lack of facilitation in patient JD might result from a deficit in the ability to generate or process the magnitude codes (Ashkenazi et al., 2008; Cohen Kadosh et al., 2008a). Either way, the significant difference in the facilitation component between JD and controls suggests a specific difficulty in processing of magnitude information. Furthermore, these differences between the facilitation and interference components stress the importance of including neutral trials when studying special populations in order to separate the components apart.

In a wider perspective, our findings contribute to the discussion regarding hemispheric lateralization of numerical processing. There is evidence that both the right and left IPS are involved in numerical representation (e.g., Ansari, 2008). However, it seems that a lesion to one hemisphere alone is enough to create a deficit in numerical processing. Specifically, the current findings suggest that a lesion to the left IPS is sufficient to lead to a deficiency in magnitude processing, both in automatic processing (i.e., the size congruity effect) and in mental representation (i.e., the distance effect). These findings are in line with previous patient studies (e.g., Lemer et al., 2003; Ashkenazi et al., 2008) but challenge TMS findings suggesting that the right, and not left IPS, is necessary for automatic processing of numerical information (Cohen Kadosh et al., 2007, 2012). To settle the dispute, several issues need to be taken into consideration.

First, it was suggested that an inter-subject variability appears in specific hemispheric lateralization (Chochon et al., 1999). Thus, both the right and left IPS play a role in magnitude processing, but some subjects rely mostly on the left or right hemisphere during magnitude processing, whereas others rely on bilateral activation. Accordingly, whereas some patients present deficits in magnitude processing following a left parietal lesion (e.g., Ashkenazi et al., 2008), others show such deficits following a right parietal lesion (e.g., Delazer and Benke, 1997). Thus, JD’s left parietal lesion might reflect such a pattern of left parietal activation during magnitude processing.

Second, whereas most patient studies suggest the left IPS is necessary for numerical processing, the role of the right IPS was implied from studies employing TMS (Cohen Kadosh et al., 2007, 2012). The differences in behavioral deficits between neuropsychological patients and healthy subjects following ‘virtual lesions’ are widely discussed in the literature (Walsh and Cowey, 1998, 2000; Davis, 2014). In the scope of the current discussion, we would like to highlight one important difference between TMS and patient studies: localization of the lesion. JD’s lesion was restricted to left parietal gray matter without involvement of white matter tracts. A similar focal lesion was described in patient AD (Ashkenazi et al., 2008), suggesting the involvement of the left parietal cortex is necessary for such processing. In contrast, recent studies suggest that stimulating a specific cortical location by TMS might actually affect anatomically distant but functionally related brain regions (Walsh and Cowey, 1998; Blankenburg et al., 2008, 2010; Feredoes et al., 2011). Thus, the TMS described in Cohen Kadosh et al.’s studies might not be restricted to the right parietal cortex but rather additional structures [possibly both left and right regions (Walsh and Cowey, 1998; Blankenburg et al., 2008, 2010; Feredoes et al., 2011)].

In the mental clock task, JD performed as accurately as controls but much slower. JD’s highly accurate performance might suggest her mental manipulation abilities remained intact. However, given the specific role of each hemisphere while carrying out the task (Formisano et al., 2002; Sack et al., 2002), it is also probable that her accurate performance is a result of neural compensation. Namely, JD’s lesion was restricted to the left posterior parietal lobe, leaving the right parietal lobe intact. Thus, we suggest that when performing the mental imagery task, the intact right parietal lobe compensated for the left parietal lobe’s deficiency, enabling both image construction and mental comparison of the images. Further studies can examine this suggestion in an imaging study. Although accurate, JD performed the mental clock task much slower than controls. Slowed processing speed was found among patients following temporal brain lesions for both auditory and visual stimuli (Peers et al., 2005). However, whereas JD performed slower in both tasks, her performance in the auditory mental clock task was four times slower than controls (vs. 1.5–2 times slower in the visual size congruity task). What might explain this difference? One major difference between the mental clock and the size congruity task is the modality in which the magnitudes to be compared are presented. In the size congruity task, participants are presented with visual displays and the working memory load is minimal during comparison. In the mental clock task, however, participants need to translate auditory stimuli into mental magnitudes, and maintain this representation in order to carry out the comparison. Thus, the mental clock task imposes much higher load on working memory. JD has presented some impairments in working memory, specifically in maintenance of information. We suggest that these impairments are more profound in the mental clock task, leading to much longer RTs in this task compared to controls. To summarize, the patient’s mildly impaired working memory abilities (as can be seen in the neuropsychological test and the slowed performance in the mental clock task) as well as a specific impairment in magnitude processing both led to abnormal performance in the mental clock task. However, we suggest that the specific impairment in the mental clock task (much steeper distance effect in the task) can be better attributed to impairment in magnitude processing and not working memory abilities.

Whereas these two tasks have marked differences in the stimuli presentations, they share the process of magnitude comparison. This process was directly examined by the distance effect. Importantly, in both tasks, JD presented a steeper distance effect compared to controls. The steeper slope of the distance effect fits with the assumption of immature numerical abilities. Previous studies described a decrease in the numerical distance effect with age; namely, younger children show a larger distance effect compared to older children (Sekuler and Mierkiewicz, 1977; Duncan and McFarland, 1980; Holloway and Ansari, 2008). Whether this child-like pattern is a result of a deficit in a domain-general comparison process (Holloway and Ansari, 2008) or in a domain-specific magnitude mechanism (Cohen Kadosh et al., 2005), the similarity between the visual and imagery tasks fits with the notion of a shared representation of magnitudes (Walsh, 2003; Cohen Kadosh et al., 2008b; Cantlon et al., 2009; Henik et al., 2012, 2017).

## Conclusion

The current findings suggest that a focal lesion to the left IPS results in immature child-like magnitude representation. Importantly, this is similar when processing symbolic visually presented numbers and when operating on mentally constructed magnitudes. This similarity is in accord with the assumption of a shared representation of magnitudes, which is not limited to numbers, and fits with previous theories on the role of the parietal lobe for linking different types of magnitude information.

## Ethics Statement

This study was carried out in accordance with the recommendations of university’s ethics committee, with written informed consent from all subjects. All subjects gave written informed consent in accordance with the Declaration of Helsinki. The protocol was approved by the Ben-Gurion University’s ethics committee.

## Author Contributions

YG and SN: conception and design of study, data acquisition, statistical analyses, interpretation of data, drafting the manuscript, editing and revising the manuscript. GI: patient recruitment and critical revision. AH: conception and design of study, and critical revision.

## Conflict of Interest Statement

The authors declare that the research was conducted in the absence of any commercial or financial relationships that could be construed as a potential conflict of interest.
