# Supplementary material for: Visual and Imagery Magnitude Comparisons Are Affected Following Left Parietal Lesion
Source: Front Psychol. 2017 Sep 19;8:1622. doi: 10.3389/fpsyg.2017.01622 (PMC5610698; doi:10.3389/fpsyg.2017.01622)
Supplement: Supplementary file 1 [file Data_Sheet_1.docx]

**Supplemental Materials**

**Visual and Imagery Magnitude Comparisons are Affected Following Left Parietal Lesion**

Yarden Gliksman, Sharon Naparstek, Gal Ifergane and Avishai Henik

* Correspondence: Yarden Gliksman; E-mail [yarden.gliksman@gmail.com](mailto:yarden.gliksman@gmail.com)

**Arithmetic Battery**

Part 1: Number comprehension and production

1. *Comparing digits.* Comparison of 8 pairs of numbers: two pairs of three-digit numbers, 3 pairs of four-digit numbers and 3 pairs of five-digit numbers. The participants had to mark a circle around the largest number.
2. *Counting forward and backward.* The participants had to count forward 7 times (e.g., count from 793-802) and backward 4 times (e.g., count from 8063-798). The initial number was a two-, three- or four-digit number.
3. *Serial order*. Participants were presented with 14 arithmetical series and had to fill the next 3 numbers in the sequence, from largest to smallest and from smallest to largest.
4. *Comparing fractions.* The participants were presented with 8 pairs of decimals and had to mark a circle around the largest number.
5. *Verbal problems*. Eight verbal problems were presented and participants were asked to: 1) Circle the correct operation for the given problem (addition, subtraction, multiplication or division). 2) Write down the representative equation. 3) Solve the equation. For example: In a building, there are 31 floors. The height of each floor is 4 meters. What is the height of the building? Answer: step 1 - the correct operation is multiplication; step 2 - the correct equation is: 31 × 4 = ; and step 3 - the correct answer is: 124.
6. *Procedural knowledge*. Participants were presented with 8 horizontal equations and were asked to copy them to vertical equations. Four equations were composed of real numbers and 4 equations were composed of decimals. The real number equations included 2, 3 or 4 numbers of one-, two-, three- or four-digit numbers. For example: 2,306+28+2+236= for the real numbers and 3.27 + 0.365 = for the decimal equations.

Part 2: Calculation

1. *Simple pure operations*. Single-digit operations were administered (10 additions, 10 subtractions, 10 divisions and 10 multiplications). Each kind of operation appeared separately.
2. *Simple mixed operations*. Single-digit operations were administered (5 additions, 5 subtractions, 5 divisions and 5 multiplications), mixed in one block in a random order. The equations had the same level of difficulty as in the pure block.
3. *Decimals*. There were four complex addition and four complex subtraction exercises requiring knowledge of decimals. The exercises were presented vertically. The numbers contained two digits after the decimal point.
4. *Estimation*. The experimenter asked participants to estimate the results of an equation (4 additions, 4 subtractions, 4 divisions and 4 multiplications), or to decide whether the result would be bigger or smaller than a specific number. For example: estimate the result of 589+426.
5. *Comparing equations.* Participants were asked to decide which equation would result in a larger number. Participants were instructed to decide according to estimation and not accurate calculation. There were 8 comparisons (2 additions, 2 subtractions, 2 divisions and 2 multiplications). For example: 71-38 or 73-28.
6. *Vertical operation.* Participants had to solve complex written arithmetic problems (addition, subtraction, multiplication, and division). The first 8 exercises were addition and subtraction, and the remaining 8 were multiplication and division. Exercises included problems with and without borrowing, using each operation.
